# Prophylactic administration of ivermectin attenuates SARS-CoV-2 induced disease in a Syrian Hamster Model

**DOI:** 10.1038/s41429-023-00623-0

**Published:** 2023-04-25

**Authors:** Takayuki Uematsu, Tomomi Takano, Hidehito Matsui, Noritada Kobayashi, Satoshi Ōmura, Hideaki Hanaki

**Affiliations:** 1 Biomedical Laboratory, Division of Biomedical Research, Kitasato University Medical Center, Kitamoto, Saitama, Japan; 2 Laboratory of Veterinary Infectious Disease, Department of Veterinary Medicine, Kitasato University, Towada, Aomori, Japan; 3 Infection Control Research Center, Ōmura Satoshi Memorial Institute, Kitasato University, Minato-ku, Tokyo, Japan; 4 Drug Discovery Project from Natural Products, Ōmura Satoshi Memorial Institute, Kitasato University, Minato-ku, Tokyo, Japan

**Keywords:** Antiviral agents, Viral infection

## Abstract

COVID-19, caused by SARS-CoV-2 infection, is currently among the most important public health concerns worldwide. Although several effective vaccines have been developed, there is an urgent clinical need for effective pharmaceutical treatments for treatment of COVID-19. Ivermectin, a chemical derivative of avermectin produced by *Streptomyces avermitilis*, is a macrocyclic lactone with antiparasitic activity. Recent studies have shown that ivermectin inhibits SARS-CoV-2 replication in vitro. In the present study, we investigated the in vivo effects of ivermectin in a hamster model of SARS-CoV-2 infection. The results of the present study demonstrate oral administration of ivermectin prior to SARS-CoV-2 infection in hamsters was associated with decreased weight loss and pulmonary inflammation. In addition, the administration of ivermectin reduced pulmonary viral titers and mRNA expression level of pro-inflammatory cytokines associated with severe COVID-19 disease. The administration of ivermectin rapidly induced the production of virus-specific neutralizing antibodies in the late stage of viral infection. Zinc concentrations leading to immune quiescence were also significantly higher in the lungs of ivermectin-treated hamsters compared to controls. These results indicate that ivermectin may have efficacy in reducing the development and severity of COVID-19 by affecting host immunity in a hamster model of SARS-CoV-2 infection.

## Introduction

The first case of COVID-19 (coronavirus disease 2019) infection caused by SARS-CoV-2 (severe acute respiratory syndrome coronavirus 2) was confirmed in China in December 2019. More than three years later, COVID-19 infection has shown no signs of abatement and has become a major public health concern around the world. COVID-19 infection is not a fulminant infection such as viral hemorrhagic fever but can progress to severe respiratory failure and thrombosis, potentially leading to death [1, 2]. In addition, cases of long-lasting sequelae such as chronic fatigue, alopecia, circulatory disturbances, and olfactory and gustatory disturbances after recovery have been reported [3, 4]. Several vaccines have been developed for the prevention of COVID-19 infection with proven efficacy [5, 6]. However, vaccines require significant development time before being readily available to all suitable recipients. Moreover, the efficacy of vaccines against certain mutant SARS-CoV-2 strains may be attenuated [7]. Studies have also shown that the protection conferred by vaccines against SARS-CoV-2 infection begins to decline several months after the second vaccination, with the risk of breakthrough infection gradually increasing from 90 days after the second vaccination [8]. In addition, despite the active global development of COVID-19 therapeutics, few drugs have demonstrated consistent efficacy. As the global spread of COVID-19 infection remains unabated, there is a need for inexpensive and safe antiviral drugs with prophylactic and therapeutic effects that can be made available worldwide independently of the medical environment.

Ivermectin, a chemical derivative of avermectin produced by *Streptomyces avermitilis*, is a macrocyclic lactone with antiparasitic activity in oral administration [9, 10]. Recent studies have shown that ivermectin inhibits the replication of SARS-CoV-2 in vitro [11]. Accordingly, clinical trials evaluating the potential of ivermectin as a COVID-19 treatment are now being conducted worldwide. However, there is currently a lack of good-quality evidence supporting the use of ivermectin for the treatment or prevention COVID-19, as demonstrated in a recent Cochrane systematic review [12].

In the present study, we evaluated the in vivo effects of ivermectin and developed a SARS-CoV-2 infection model using Syrian hamsters, which are a popular rodent model for COVID-19 [13–15] to determine the prophylactic effects of ivermectin administration on COVID-19. Ivermectin was administered prophylactically to determine the therapeutic efficacy of ivermectin in Syrian hamsters infected with SARS-CoV-2.

## Materials and methods

### Hamsters

Three-week-old Male Syrian hamsters were purchased from Japan SLC, Inc. (Hamamatsu, Japan) and used for experiments after a week of quarantine. Hamsters were housed under specific pathogen-free conditions. Animal experiments were conducted according to the protocol approved by the president of Kitasato University after the review by the Institutional Animal Care and Use Committee (approval number: 2020-7).

### Reagents

Ethanol, polyethylene glycol (PEG) 400, and hydrochloric acid were purchased from Fujifilm Wako Pure Chemical (Osaka, Japan). Tween 80 was purchased from Kanto Chemical (Tokyo, Japan). Metallo Assay Zinc LS was purchased from Metallogenics (Chiba, Japan). Hamster D-Dimer (D2D) ELISA kits were purchased from My BioSource (San Diego, CA). Ivermectin was purchased from Sigma Aldrich (St. Louis, MO).

### SARS-CoV-2 Virus

The SARS-CoV-2 strain, JPN/Kanagawa/KUH003 (accession # LC630936) isolated from patients with COVID-19 admitted to Kitasato University Hospital, was used in this experiment [16]. This study was approved by the Kitasato University Medical Ethics Organization (KMEO; approval numbers: KMEO B20-016-1, KMEO B20-016-2).

### SARS-CoV-2 infection in hamsters

Young male Syrian hamsters were orally administered ivermectin prepared with 5% ethanol (w/v), 10% polyethylene glycol (PEG) 400 (w/v), and 5% Tween 80 (w/v) at a concentration of 1 mg kg^−1^ 6 h prior to inoculation. After 6 h, hamsters were anesthetized with medetomidine hydrochloride, midazolam, and butorphanol tartrate and infected with 5 × 10^4^ PFUs of SARS-CoV-2 (JPN/Kanagawa/KUH003) by intranasal administration.

### Virus titers

Lung tissues were collected at 0, 3, 6, and 10 days after SARS-CoV-2 infection and homogenized using a gentleMACS dissociator (Miltenyi Biotec, Bergisch Gladbach, Germany). Lung homogenates were serially diluted with D-MEM (Fujifilm Wako Pure Chemical) supplemented with 2% foetal bovine serum (Life Technologies, Carlsbad, CA) and antibiotics (100 IU ml^−1^ penicillin and 100 μg ml^−1^ streptomycin; Fujifilm Wako Pure Chemical). For plaque assays, VeroE6/TMPRSS2 cells (obtained from JCRB cell bank, Osaka, Japan) were plated at 4 × 10^5^ cells in a flat-bottomed 6-well plate (Corning, Corning, NY) a day before infection. Supernatants from serially diluted lung homogenates were used to infect VeroE6/TMPRSS2 cells at 37 °C for 1 h. Cells were subsequently overlaid with D-MEM mixed with 1% carboxymethyl cellulose sodium (Fujifilm Wako Pure Chemical). Plaques were visualized by staining with methylene blue (Nacalai tesque, Kyoto, Japan), and counted 3 days after infection.

### Histology

Hamsters were euthanized by intraperitoneal administration of sodium pentobarbital at 0, 3, 6, and 10 days after SARS-CoV-2 infection and lungs were collected. Paraffin embedding and hematoxylin and eosin staining of tissues were performed using standard methodologies.

### qPCR analysis for mRNA expression

Total RNA was extracted from cells using the ISOGEN II (NIPPON GENE, Tokyo, Japan). cDNA was synthesized from mRNA using One Step PrimeScript III RT-qPCR Mix with UNG (Takara Bio, Shiga, Japan). RT products were directly subjected to qPCR in a 7500 real-time PCR system (Applied Biosystems, Foster City, CA, USA) using Syrian hamster-specific primers and probes designed at Takara Bio (Supplementary Table 1).

### Zinc concentration measurements

After adding hydrochloric acid to lung homogenates to form an acidic mixture at ~0.1 M, the zinc concentration was determined using the Metallo Assay Zinc LS according to the manufacturer’s instructions.

### Neutralizing antibody titer measurements

Plasma was serially twofold diluted in medium and mixed with an equal volume of a virus suspension containing approximately 200 PFU/100 µl. Mixtures were incubated at 37 °C for 1 h. Cells were then inoculated in 96-well flat-bottomed plates and incubated in an atmosphere of 5% CO_2_ in air at 37 °C for 3 days. For each serum dilution, tests were duplicated. Neutralizing antibody titers were expressed as a reciprocal of the highest dilution of the test sera that inhibited the cytopathic effect completely.

### Statistical analysis

Statistical analyses were performed using GraphPad Prism (GraphPad, San Diego, CA, USA). Student’s t-test was used to evaluate differences between datasets. Values are presented as means ± SEM. *P* values less than 0.05 were considered statistically significant.

## Results

### Prophylactic administration of ivermectin prevents weight loss in a hamster model of SARS-CoV-2 infection

**Fig. 1:**
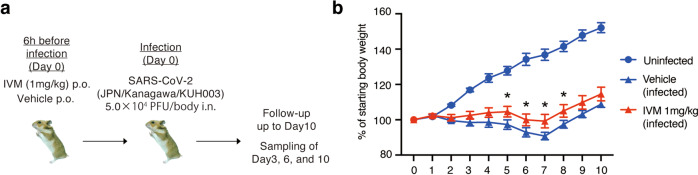
Prophylactic administration of ivermectin prevents weight loss in a hamster model of SARS-CoV-2 infection. **a** Schematic of the experimental design. **b** Bodyweight change in Syrian hamsters following SARS-CoV-2 infection. Both vehicle-treated and ivermectin (IVM)-treated hamsters (*n* = 6 per group) were infected by intranasal injection of 5 × 10^4^ plaque-forming units (PFU) of SARS-CoV-2 followed by assessments for bodyweight change. Hamsters without viral infection, *n* = 3 per group. Data are presented as the means ± SEM. *P < 0.05 by Student’s *t* test

We preliminarily investigated ivermectin administration routes and dosages by determining the dose required to inhibit weight loss after SARS-CoV-2 infection in a hamster model. The clinical dose of ivermectin in humans is 150–400 µg kg^−1^ [9, 10]. Therefore, hamsters were infected with SARS-CoV-2 after administration of a single dose of ivermectin at 250 µg kg^−1^ or 500 µg kg^−1^. However, an inhibitory effect on weight loss after SARS-CoV-2 infection was not observed at these concentrations with oral or intraperitoneal administration (Supplementary Fig. 1a, b). Based on the results of the preliminary study, ivermectin was prepared with 5% ethanol (w/v), 10% polyethylene glycol (PEG) 400 (w/v), and 5% Tween 80 (w/v) at a concentration of 1 mg kg^−1^ 6 h prior to inoculation. We then inoculated young male Syrian hamsters intranasally with 5 × 10^4^ plaque forming units (PFUs) of SARS-CoV-2 (JPN/Kanagawa/KUH003) isolated from COVID-19 patient specimens (Fig. 1a) [16]. Analysis of body weight changes after inoculation demonstrated that hamsters infected with SARS-CoV-2 had temporary weight loss which peaked on the 7th day of infection. However, weight loss was suppressed in hamsters orally administrated ivermectin prior to inoculation (Fig. 1b). These results indicate that oral administration of 1 mg kg^−1^ ivermectin in hamsters acts prophylactically against SARS-CoV-2 infection and prevents weight loss after viral infection.

### Prophylactic administration of ivermectin reduces pulmonary disease in a hamster model of SARS-CoV-2 infection

**Fig. 2:**
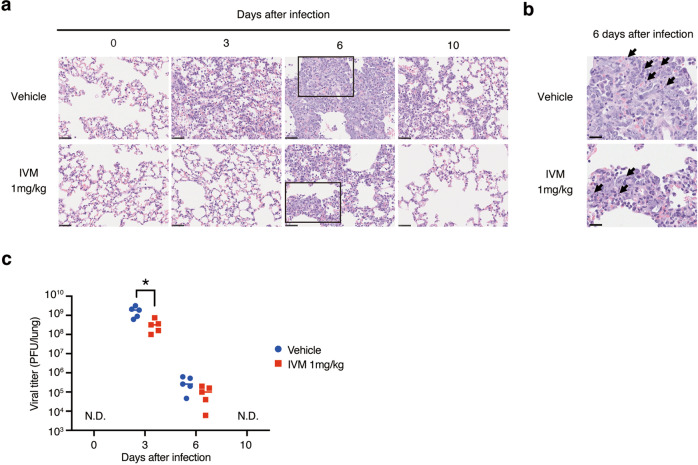
Prophylactic administration of ivermectin reduces the severity of pulmonary disease in a hamster model of SARS-CoV-2 infection. **a** Hematoxylin and eosin staining of the lungs on days 0, 3, 6 and 10 after SARS-CoV-2 infection. Bar = 50 μm. **b** Magnified image of hematoxylin and eosin staining of lungs 6 days after SARS-CoV-2 infection; the area enclosed by the square in (**a**) is magnified. Bar = 25 μm.　**c** Viral titers in infected lungs. Lungs from vehicle-treated or IVM-treated hamsters infected with SARS-CoV-2 on days 0, 3, 6 and 10 (*n* = 5 per group) were homogenized and virus titers were quantified by plaque assay in VeroE6/TMPRSS2 cells. N.D., not detected. Data are presented as the means ± SEM. *P < 0.05 by Student’s t-test

We next examined the pharmacological effects of ivermectin in the lungs, the primary target of SARS-CoV-2 infection. We observed pulmonary changes associated with viral infection on histopathological examination of hamster lungs on days 3 and 6 of SARS-CoV-2 infection, including thickening of alveolar walls and infiltration of neutrophils. However, ivermectin-treated hamsters had decreased pulmonary inflammation (Fig. 2a, b). Furthermore, we compared pulmonary virus titers with plaque assays using SARS-CoV-2-sensitive VeroE6/TMPRSS2 cells [17]. Viral titers in the lungs of ivermectin-treated hamsters were reduced by a factor of 10 compared to vehicle-treated hamsters on days 3 after infection (Fig. 2b). These results indicate that prophylactic administration of ivermectin reduces pulmonary injury and viral titers in a hamster model of SARS-CoV-2 infection.

### Prophylactic administration of ivermectin inhibits pulmonary expression of inflammatory cytokines in response to SARS-CoV-2 infection

**Fig. 3:**
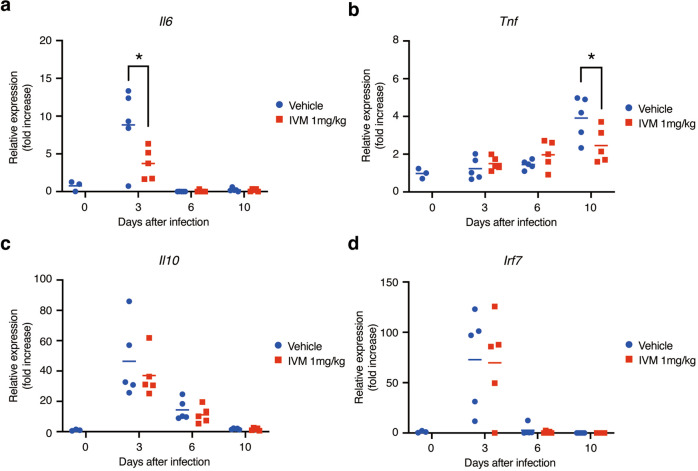
Prophylactic administration of ivermectin inhibits pulmonary inflammatory cytokine expression following SARS-CoV-2 infection. **a** *Il6*, (**b**) *Tnf*, (**c**) *Il10*, and (**d**) *Irf7* mRNA expression levels in lungs of vehicle-treated or IVM-treated hamsters (*n* = 5 per group) after SARS-CoV-2 infection. Data are presented as the means ± SEM of duplicates. *P < 0.05 by Student’s t-test

When SARS-CoV-2 infects target cells, viral genomes are detected by viral sensors such as RIG-I, MDA-5, and TLR3 which then induce host innate immune responses [18, 19]. Although activation of an appropriate innate immune response is essential for viral elimination, severe cases of COVID-19 are reported to have excessive activation of innate immunity [1, 2]. Inappropriate activation of innate immunity leads to excessive production of inflammatory cytokines, which are often lethal to the host. Therefore, quantitative PCR was performed using Syrian hamster-specific primers and probes to compare mRNA expression levels of several cytokines and interferon-inducible transcription factors in the lung (Supplementary Table 1). Expression of pro-inflammatory cytokines, such as *Il6* in the early stage of viral infection and *Tnf* in the late stage of viral infection, were decreased in ivermectin-treated hamsters (Fig. 3a, b). On the other hand, expression of the immunosuppressive cytokine, *Il10*, or the interferon-inducible transcription factor, *Irf7*, remained unchanged (Fig. 3c, d). These results suggest that ivermectin suppresses the expression of inflammatory cytokines associated with SARS-CoV-2 infection at the genetic level.

### Prophylactic administration of ivermectin reduces the severity of pathological changes associated with SARS-CoV-2 infection

**Fig. 4:**
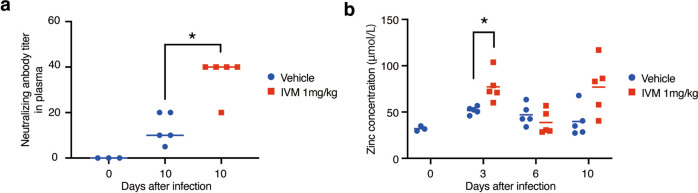
Prophylactic administration of ivermectin reduces the severity of pathological changes associated with SARS-CoV-2 infection. **a** Plasma titers of neutralizing antibodies on day 0 and 10 in vehicle-treated or IVM-treated hamsters (*n* = 5 per group) infected with SARS-CoV-2. Neutralizing antibody titers are expressed as a reciprocal of the highest dilution of the test plasma that completely inhibited the cytopathic effect. **b** Zinc concentrations in lungs from vehicle-treated or IVM-treated hamsters (*n* = 5 per group) after SARS-CoV-2 infection. Data are presented as the means ± SEM of duplicates. *P < 0.05 by Student’s t-test

Ivermectin has been reported to have a variety of pharmacological effects on the body, including anticancer and antiviral activities in addition to anthelmintic activity [20, 21]. Ivermectin has been reported to have in vitro antiviral activity against HIV and dengue virus [22–24]; however, the in vivo antiviral activity of ivermectin remains unclear. In severe cases of COVID-19, elevated plasma D-Dimer levels and decreased zinc levels have been reported [25]. Efficient induction of neutralizing antibodies is crucial for viral elimination [26]. We therefore examined plasma D-Dimer levels in SAR-CoV-2-infected hamsters prophylactically treated with ivermectin and found no clear difference compared to the vehicle-treated group (Supplementary Fig. 2). We further measured virus neutralizing antibody titer in plasma on 10 day after infection, which we considered the recovery period. Plasma titers of neutralizing antibodies were significantly higher in ivermectin-treated hamsters compared to vehicle-treated hamsters (Fig. 4a). In addition, ivermectin-treated animals had higher zinc concentrations in lung suspensions on day 3 after infection, with a similar trend observed 10 days after infection (Fig. 4b). These results suggest that prophylactic administration of ivermectin has multiple effects that lead to a reduction in the severity of pathological changes associated with SARS-CoV-2 infection.

## Discussion

In the present study, we demonstrate the efficacy of prophylactic administration of ivermectin in the protection against SARS-CoV-2 infection in a Syrian hamster model. Hamsters orally administered ivermectin prior to infection with SARS-CoV-2 had reduced body weight loss (Fig. 1) and reduced viral titers and inflammation in the lungs, a major target organ of SARS-CoV-2 (Fig. 2). Furthermore, prophylactic administration of ivermectin suppressed the expression of pro-inflammatory cytokines (IL-6 and TNF) reported to be increased in severe cases of COVID-19 (Fig. 3). Moreover, prophylactic administration of ivermectin increased plasma titers of neutralizing antibodies and pulmonary zinc levels leading to suppression of severe COVID-19 (Fig. 4).

We first conducted a preliminary study of the dosage and administration of ivermectin in hamsters as described above. There are several reports of the efficacy of ivermectin administration against coronavirus infection [27, 28]; however, we observed no protective effect against SARS-CoV-2 at ivermectin doses lower than 1 mg kg^−1^. The common dose of ivermectin in human clinical practice is 200 µg kg^−1^, a lower dose than required for efficacy against SARS-CoV-2 infection in our hamster model. This difference in the concentration of ivermectin required for efficacy between previous reports and our study may be due to differences in the solvent used for administration or the sex or age of hamsters used. Therefore, the clinical application of ivermectin as a therapy for COVID-19 in humans appears to require careful investigation in clinical trials in addition to in vivo studies to determine the most effective dosage, frequency and administration methods. Although multiple clinical trials on ivermectin against human COVID-19 have been conducted globally, several studies deny the effect of this compound. Therefore, guidelines such as the previously mentioned Cochrane Review do not recommend the use of ivermectin for COVID-19.　We also unambiguously underline that our present study reports only results obtained in an animal model and does not intend to promote arbitrary ivermectin abuse based on self-determination.

However, prophylactic ivermectin administration certainly reduces COVID-19-related pathology in animal models. Therefore, we believe that ivermectin and its derivatives exhibit therapeutic potential as candidates for treating COVID-19. Different immunomodulatory effects have been reported not only for ivermectin but also for other macrolide compounds (e.g., erythromycin and azithromycin) [29–32]. The mechanism of action of ivermectin in protecting against SARS-CoV-2 infection is currently being investigated in detail using cultured cells and rodent models such as human ACE2 (angiotensin converting enzyme 2) transgenic mice and hamsters. Although we were unable to demonstrate a clear mechanism of action in the present study, we posit the activity of ivermectin is mediated by host immunity as ivermectin is effective when administered as a single dose before viral infection. In addition to activating parasite-specific glutamine-gated chloride channels, ivermectin has been reported to have multiple bioactivities, including inhibition of the interaction between importin α/β and RNA virus component proteins, and structural stabilization of the calcium-activated channel, P2X4 receptor [33]. Of note, an increase in virus-specific neutralizing antibody titers and pulmonary zinc levels following ivermectin administration were observed in the present study (Fig. 4). Changes in intracellular concentrations of divalent metal ions such as zinc and calcium reportedly affect the host immune response [34–37]. It has been reported that a high intracellular zinc level suppresses the expression of major histocompatibility complex (MHC) genes as well as co-stimulatory molecules in dendritic and CD4 positive T-helper cells during T cell-mediated immune responses [38]. Ivermectin interacts with various ion channel proteins to regulate intracellular ion concentrations, suggesting ion-mediated immunomodulatory mechanisms are involved in immune responses to SARS-CoV-2 infection. Recently, it has been suggested that tissue expression of ACE2, the receptor for SARS-CoV-2, in the lungs and gastrointestinal tract is regulated by signaling through the nuclear receptor FXR (farnesoid X receptor), and thus FXR signaling may be a potential therapeutic target for COVID-19 [39]. In this regard, it has been reported that ivermectin binds to FXR with high affinity and negatively regulates lipid and cholesterol metabolism in a mouse in vivo model [40]. This suggests that ivermectin may downregulate the expression of ACE2 on SARS-CoV-2-sensitive cells in an FXR signaling-dependent manner by binding to FXR. In our present study, prophylactic administration of ivermectin effectively reduced the pathogenesis associated with SARS-CoV-2 infection, which may be due to such FXR signaling-dependent effects *via* ivermectin.

In conclusion, we demonstrate the efficacy of prophylactic administration of ivermectin in a hamster model of SARS-CoV-2 infection. Ivermectin and its derivatives may represent an effective therapeutic option for COVID-19; however, further in vitro and in vivo analyses are required to determine the optimal dose and method of administration of ivermectin. High quality clinical trials are required to fully elucidate the mechanism of action of ivermectin and validate the potential of ivermectin as a therapeutic option for the prevention and treatment of COVID-19.

## Supplementary information

Supplementary Information
