# Supplementary material for: Prophylactic administration of ivermectin attenuates SARS-CoV-2 induced disease in a Syrian Hamster Model
Source: J Antibiot (Tokyo). 2023 Apr 25;76(8):481–8. doi: 10.1038/s41429-023-00623-0 (PMC10127164; doi:10.1038/s41429-023-00623-0)
Supplement: Supplementary file 1 — Supplementary Information [file 41429_2023_623_MOESM1_ESM.docx]

**Supplementary Information**

**Prophylactic Administration of Ivermectin Attenuates SARS-CoV-2 Induced Disease in a Syrian Hamster Model**

**Takayuki Uematsu^1^*, Tomomi Takano^2^, Hidehito Matsui^3^, Noritada Kobayashi^1^, Satoshi Ōmura^4^, Hideaki Hanaki^3^**

^1^Biomedical Laboratory, Division of Biomedical Research, Kitasato University Medical Center, Kitamoto, Saitama, Japan.

^2^Laboratory of Veterinary Infectious Disease, Department of Veterinary Medicine, Kitasato University, Towada, Aomori, Japan.

^3^Infection Control Research Center, Ōmura Satoshi Memorial Institute, Kitasato University, Minato-ku, Tokyo, Japan.

^4^Drug Discovery Project from Natural Products, Ōmura Satoshi Memorial Institute, Kitasato University, Minato-ku, Tokyo, Japan.

*Correspondence and requests for materials should be addressed to Takayuki Uematsu (email: [tuematsu@insti.kitasato-u.ac.jp](mailto:tuematsu@insti.kitasato-u.ac.jp)).

**a**

**b**

**Supplementary Figure 1.**

**A Preliminary Study on the Effective Dose of Ivermectin against SARS-CoV-2 Infection in a Hamster Model.** Bodyweight change of Syrian hamsters after SARS-CoV-2 infection in male hamsters treated with (**a**) 250 or (**b**) 500 μg/kg of ivermectin. Both vehicle- and IVM-treated hamsters (n = 3 per group) were infected by intranasal injection with 5 × 10^4^ plaque-forming units (PFU) of SARS-CoV-2, followed by the assessment of bodyweight changes. Uninfected hamster sample numbers were n = 3 per group. Data are presented as means ± SEM. *P < 0.05 by the Student’s t-test.

**Supplementary Figure 2.**

**The D-Dimer concentration of plasma in a hamster SARS-CoV-2 infection model.** The D-Dimer concentration in plasma of vehicle or IVM-treated hamsters (n = 5 per group) after SARS-CoV-2 infection. Data are presented as the means ± SEM of duplicates. *P < 0.05 by the Student’s t test.

| Target gene | Primer or probe | Sequence (5' to 3') |
| --- | --- | --- |
| *Il6* | Forword primer | GAGACGAACAATGTACAAGATAACAA |
|  | Reverse primer | CGTGACTATTTTATCTGGACCCTTTA |
|  | Internal probe | [6FAM]ACAAAGCCAGAGTCATTCAGAGCACCATCA[TAMRA] |
| *Tnf* | Forword primer | GTCCTACGAGGATAACGTCAACC |
|  | Reverse primer | TCGGCACTGAGTCGGTCAC |
|  | Internal probe | [6FAM]CATCAAGAGCCCCTGCCCCAAGGAAAC[TAMRA] |
| *Il10* | Forword primer | CATCGATTTCTCCCCTGTGAAAA |
|  | Reverse primer | GCCTTTCTCTTGGAGCTTATTAAAG |
|  | Internal probe | [6FAM]ATCCTTCACCTGTTCCACAGCCTTGCTT[TAMRA] |
| *Irf7* | Forword primer | AGGCGACAAGGATCACCACA |
|  | Reverse primer | GCACTCCCTCCAGGTGAGC |
|  | Internal probe | [6FAM]CATGGCTCCAACTTGACCAGAATCAGGCTC[TAMRA] |
| *Gapdh* | Forword primer | CCTTCATTGACCTCAACTACATGG |
|  | Reverse primer | GCTCCTGGAAGATGGTGATGG |
|  | Internal probe | [6FAM]ACTCGGCACCAGCATCACCCCATTTG[TAMRA] |

**Supplementary Table 1.**

**Detailed sequence information of the primers and probes for qRT-PCR.** Primers and probes for Syrian hamsters were designed at Takara Bio. The primers and probes are listed on above.
